# Exosomes from NSC-34 Cells Transfected with hSOD1-G93A Are Enriched in miR-124 and Drive Alterations in Microglia Phenotype

**DOI:** 10.3389/fnins.2017.00273

**Published:** 2017-05-17

**Authors:** Sara Pinto, Carolina Cunha, Marta Barbosa, Ana R. Vaz, Dora Brites

**Affiliations:** ^1^Neuron Glia Biology in Health and Disease, Research Institute for Medicines (iMed.ULisboa), Faculty of Pharmacy, Universidade de LisboaLisbon, Portugal; ^2^Department of Biochemistry and Human Biology, Faculty of Pharmacy, Universidade de LisboaLisbon, Portugal

**Keywords:** amyotrophic lateral sclerosis, microglia polarization, microglia surface receptors, inflamma-microRNA, motor neurons (MNs), MN-derived exosomes, mutant SOD1

## Abstract

Amyotrophic lateral sclerosis (ALS) is a fatal adult-onset neurodegenerative disorder affecting motor neurons (MNs). Evidences indicate that ALS is a non-cell autonomous disease in which glial cells participate in both disease onset and progression. Exosomal transfer of mutant copper-zinc superoxide dismutase 1 (mSOD1) from cell-to-cell was suggested to contribute to disease dissemination. Data from our group and others showed that exosomes from activated cells contain inflammatory-related microRNAs (inflamma-miRNAs) that recapitulate the donor cell. While glia-derived exosomes and their effects in neurons have been addressed by several studies, only a few investigated the influence of motor neuron (MN)-derived exosomes in other cell function, the aim of the present study. We assessed a set of inflamma-miRs in NSC-34 MN-like cells transfected with mutant SOD1(G93A) and extended the study into their derived exosomes (mSOD1 exosomes). Then, the effects produced by mSOD1 exosomes in the activation and polarization of the recipient N9 microglial cells were investigated. Exosomes in coculture with N9 microglia and NSC-34 cells [either transfected with either wild-type (wt) human SOD1 or mutant SOD1(G93A)] showed to be transferred into N9 cells. Increased miR-124 expression was found in mSOD1 NSC-34 cells and in their derived exosomes. Incubation of mSOD1 exosomes with N9 cells determined a sustained 50% reduction in the cell phagocytic ability. It also caused a persistent NF-kB activation and an acute generation of NO, MMP-2, and MMP-9 activation, as well as upregulation of IL-1β, TNF-α, MHC-II, and iNOS gene expression, suggestive of induced M1 polarization. Marked elevation of IL-10, Arginase 1, TREM2, RAGE, and TLR4 mRNA levels, together with increased miR-124, miR-146a, and miR-155, at 24 h incubation, suggest the switch to mixed M1 and M2 subpopulations in the exosome-treated N9 microglial cells. Exosomes from mSOD1 NSC-34 MNs also enhanced the number of senescent-like positive N9 cells. Data suggest that miR-124 is translocated from the mSOD1 MNs to exosomes, which determine early and late phenotypic alterations in the recipient N9-microglial cells. In conclusion, modulation of the inflammatory-associated miR-124, in mSOD1 NSC-34 MNs, with potential benefits in the cargo of their exosomes may reveal a promising therapeutic strategy in halting microglia activation and associated effects in MN degeneration.

## Introduction

Since the beginning of the last decade, exosomes, and their role in the central nervous system (CNS), namely in the pathophysiology of neurodegenerative diseases such as amyotrophic lateral sclerosis (ALS), have been of increased interest in the science community. Indeed, autophagy and release of extracellular vesicles (such as exosomes and microvesicles) have been pointed to be involved in the secretion of harmful/damaged proteins and RNAs to alleviate intracellular stress conditions and sustaining cell homeostasis (Baixauli et al., 2014). Once exosomes represent a new way of long distance transfer of biological molecules into other cells, they are believed to be key players in disease dissemination, as well as a powerful tool for delivering medicines (Aryani and Denecke, 2016; Budnik et al., 2016). Exosomes and microvesicles or ectosomes, originated from endosomal, and plasma membrane, respectively, contain proteins, lipids, soluble factors, mRNAs and microRNAs (miRNAs) (Brites and Fernandes, 2015).

In familial ALS (fALS), transfer of misfolded and mutant copper-zinc superoxide dismutase 1 (mSOD1) from cell-to-cell was evidenced to be mediated by exosomes (Silverman et al., 2016). Among the several potential pathogenic genes in fALS and sporadic cases (sALS), the most frequent are *C9orf72* (40% of fALS and 5–6% of sALS cases) and *SOD1* (20% of fALS and 3% of sALS cases) (Kruger et al., 2016). This fatal and progressive neurodegenerative disease affects motor neurons (MNs) in the spinal cord and motor cortex. However, neuroinflammation and peripheral immune system activation were shown to accompany ALS neurodegeneration (Zondler et al., 2017). The underlying mechanisms are still unknown, but seem to involve multiple neural cell dysfunctional processes and complex multisystem deregulation, what turns difficult the identification of specific targets and the development of successful therapies. Lately, the interplay between MNs and glial cells mediated by exosomes was suggested to be crucial in the disease outcome and progression. Actually, it was shown that astrocyte-derived exosomes may transfer mSOD1 to MNs contributing to neurodegeneration and disease spread (Basso et al., 2013). More recently, it was demonstrated that both mSOD1 and misfolded wild-type (wt) SOD1 from NSC-34 MN-like cells are transferred on the surface of exosomes and delivered to neighboring MN cells by macropinocytosis (Grad et al., 2014b).

While glia-derived extracellular vesicles and their load effects in neurons have been recently evaluated as a novel form of communication in the brain (Schiera et al., 2015; Basso and Bonetto, 2016), only a few studies have investigated the influence of MN-derived exosomes in other cell function. Such studies have demonstrated how exosomes shuttle proteins from neurons to muscle cells. Indeed, the transfer of Synaptotagmin 4 (Syt4), a membrane trafficking protein implicated in the retrograde signal, from presynaptic compartments to postsynaptic muscle cells, was evidenced to be mediated by exosomes (Korkut et al., 2013). Other studies showed that extracellular vesicles from muscle have significant effects on the survival and neurite outgrowth of NSC-34 MN-like cells (Madison et al., 2014). In addition, exosome transfer of amyloid-β (Aβ) peptide from neurons to microglia revealed to be facilitated by phosphatidylserine recognition and to be followed by transportation to lysosomes and degradation, thus decreasing the extracellular levels of Aβ (Yuyama et al., 2012). Macropinocytosis may also be involved in the internalization of exosomes by a subset of microglia, as recently observed for exosomes secreted by oligodendrocytes, in an immunologically “silent” manner (Fitzner et al., 2011).

Microglia was reported to have reduced neuroprotective properties and increased neurotoxic potential in ALS, and diverse microglia subpopulations were shown to coexist (Gerber et al., 2012; Brites and Vaz, 2014). It was considered that microglia display the M2 anti-inflammatory phenotype at the early stages of the disease, switching to the M1 classically activated subtype as the disease progresses (Zhao et al., 2013). It was also suggested that microglia acquire a unique phenotype in ALS, not directly related with M1 or M2 polarization, and show an impaired function at the end-stage of ALS disease (Chiu et al., 2013; Nikodemova et al., 2014). Understanding the underlying conditions triggering different microglia phenotype profiling may help in the development of novel therapeutic strategies directed to specific ALS disease-stages. Emerging data indicate that activation of microglia into the M1 subtype is associated with the increased expression of inflammation-related miRNAs (inflamma-miRs), secretion of proinflammatory cytokines and alarmins, such as the high mobility group box protein 1 (HMGB1), and release of exosomes (Brites and Fernandes, 2015; Cunha et al., 2016; Falcão et al., 2017). Several microglial receptors have been implicated in the generation of an inflammatory response, like the Receptor for Advanced Glycation Endproducts (RAGE) by binding HMGB1 or the toll-like receptor-4 (TLR4) by binding lipopolysaccharide (LPS) and Aβ peptide. Others, in promoting phagocytosis and inhibiting the production of inflammatory mediators, such as the triggering receptor expressed by myeloid cells 2 (TREM2) (Doens and Fernandez, 2014). Such receptors were shown to play important roles in microglial activation status.

Recent evidences showed that endogenous miR-155 and miR-146a, two critical miRNAs in regulating inflammation, are present in exosomes and pass between immune cells, such as dendritic cells, intervening in the cellular inflammatory response (Alexander et al., 2015). MiRNAs are a subset of non-coding RNAs that may inhibit mRNA translation, cause mRNA degradation or even switch from repression to activation depending on cell cycle stages (Kye and Goncalves Ido, 2014; Li et al., 2015). Presynaptic alterations were shown to correlate with increased miR-124 and miR-142, which regulate Rab3a expression in motor nerves providing the basis for impaired synaptic function in neuromuscular disorders (Zhu et al., 2013). Dysregulation of miRNA expression and function in ALS also include elevation of miR-155 in either the rodent ALS model or in the spinal cord tissue of both fALS and sALS patients (Koval et al., 2013). In this study it was demonstrated that inhibition of miR-155 extended the survival of animals. Consistent with these results, ablation of miR-155 in the SOD1 mouse recovered microglia function and attenuated the disease (Butovsky et al., 2015). In this context, the anti-miR-155 MRG-107 compound is being developed with the proposal of reducing neuroinflammation in ALS patients (http://www.alsa.org/news/media/press-releases/miragen-therapeutics-060216.html).

Despite the current knowledge on the role of exosomes from NSC-34 MNs overexpressing human SOD1 mutated in G93A in cell-to-cell transfer of mSOD1 toxicity, it was never investigated how the uptake of such exosomes by receptor cells, such as microglia, may contribute to their activation and/or loss of function. In this study we hypothesized that exosomes can transfer specific inflammatory-related miRNAs as signaling cues for N9-microglia stimulation. Here we evaluated: (i) a specific set of inflamma-miRs in mSOD1 NSC-34 like MNs (mutated in G93A) and in their derived exosomes; (ii) the preferential cell distribution of mSOD1 MN-derived labeled exosomes in a coculture system of NSC-34 MNs and N9 microglial cells; and (iii) alterations in microglia polarization and function by interaction with mSOD1 MN-derived exosomes. Increased content in miR-124 was found in mSOD1 NSC-34 MN-derived exosomes. In the coculture system of MNs and N9 microglial cells, exosomes were preferentially internalized by N9 cells. The results obtained demonstrate that exosomes from mSOD1 MNs induce early activation of inflammatory signaling pathways and delayed increased expression of miR-124, miR-146a, and miR-155, together with mixed M1 and M2 phenotypic markers. In addition, exosomes caused microglia dysfunction evidenced by the loss of phagocytic ability and increased number of senescent-like cells. Data highlight that increased level of miR-124 in circulating exosomes may reveal a good biomarker of MN degeneration in ALS and that its modulation may have benefits in halting exosomal-inflamma-miRs dissemination and induced effects on microglia activation and dysfunction.

## Materials and methods

### NSC-34 cell culture

We used mouse MN-like NSC-34 cells stably expressing wt and mSOD1, which were a kind gift from Júlia Costa (ITQB, Universidade Nova de Lisboa, Portugal). Mouse NSC-34 is a hybrid cell line produced by the fusion of MNs from the spinal cord embryos with N18TG2 neuroblastoma cells (Cashman et al., 1992) that exhibit properties of MNs after differentiation and maturation protocols. Thus, NSC-34 cells were grown in proliferation media [Dulbecco's modified Eagle's medium (DMEM) high glucose with glutamine, w/o pyruvate, supplemented with 10% of fetal bovine serum (FBS) and 1% of Penicillin/Streptomycin] and selection was made with Geneticin 418 sulfate (G418) at 0.5 mg/ml (Vaz et al., 2015). Medium was changed every 2–3 days. Culture plates were coated with Poly-d-Lysine (10 μg/ml) before plating the cells. Cells were seeded at a concentration of 5 × 10^4^ cells/ml and maintained at 37°C in a humidified atmosphere of 5% CO_2_. After 48 h in proliferation medium, differentiation was induced by changing medium for DMEM-F12 plus 1% of FBS-exosome depleted, 1% of non-essential amino acids (NEAA), 1% of Penicillin/Streptomycin and 0.5 mg/ml of G418, as indicated by Cho et al. (2011). Cells were maintained in culture with differentiation medium for 4 days *in vitro* (DIV) to induce SOD1 accumulation (Vaz et al., 2015). DMEM, DMEM-F12, FBS, Penicillin/Streptomycin, and NEAA were purchased from Biochrom AG (Berlin, Germany). G418 was obtained from Gibco/Calbiochem (Darmstadt, Germany), and Poly-d-Lysine and RPMI were from Sigma-Aldrich (St. Louis, MO, USA).

### Exosome isolation and characterization

Exosomes were isolated from the extracellular media of wt and mSOD1 NSC-34 cells, as currently in use in our lab (Cunha et al., 2016). Briefly, the culture media (20 ml) of the NSC-34 cells differentiated for 4 DIVs was centrifuged at 1,000 g for 10 min to remove cell debris. Then, the supernatant was transferred to another tube and centrifuged again at 16,000 g for 60 min, to separate microvesicles (size ~1,000 nm). The recovered supernatant was subsequently filtered in a 0.22 μm pore filter, and further centrifuged in the Ultra L-XP100 centrifuge (Beckman Coulter Inc., California, USA) at 100,000 g for 120 min to pellet exosomes (size ~100 nm). The pellet of exosomes was then resuspended in phosphate-buffered saline (PBS) and centrifuged one last time at 100,000 g for 120 min, in order to wash the pellet. All centrifugations were performed at 4°C. Characterization of exosomes in terms of size and concentration was performed by Nanoparticle tracking analysis (NTA) using the Nanosight, model LM10-HSBF (Malvern, UK) and the NTA software version 3.1. Transmission electron microscopy (TEM) technique used the Jeol JEM 1400 Transmission Electron Microscope (Peabody, MA, USA). Western blot analysis was performed as usual in our lab (Vaz et al., 2015) to evaluate the expression of alix, flotillin-1 and CD63 by using 20 μg of total protein and specific antibodies (mouse anti-Alix, Cell Signaling, #2171; mouse anti-flotillin-1, BD Biosciences, #6108; goat anti-CD63, Santa Cruz Biotechnology, #sc-31214). Normalization was made by using Amido Black staining as loading control. To evaluate total RNA and microRNA, the final pellet containing exosomes was resuspended in lysis buffer, and exosomal RNA extracted with the miRCURY Isolation Kit-Cell (Exiqon), as described below.

### N9 cell culture

Mouse microglial N9 cell line, a popular retroviral-immortalized cell line resulting from the immortalization of microglia isolated from the cortex of CD1 mouse embryos (Righi et al., 1989), was a gift from Teresa Pais (Institute Gulbenkian de Ciência, Oeiras, Portugal). This cell line shows diverse features similar to microglia in primary cultures, such as migration, phagocytosis and inflammation-related features (Fleisher-Berkovich et al., 2010). Indeed, N9 cells were shown to respond similarly to primary microglial cells derived from the same mouse strain, when treated with LPS (Nikodemova and Watters, 2011), as recently demonstrated by us (Cunha et al., 2016). Cells were cultured in Roswell Park Memorial Institute (RPMI) medium supplemented with 10% of FBS, 1% of L-glutamine (Biochrom AG) and 1% of Penicillin/Streptomycin. Cells were seeded at a concentration of 1 × 10^5^ cells/ml and maintained at 37°C in a humidified atmosphere of 5% CO_2_.

### Coculturing of NSC-34 with N9 microglial cells, exosomal labeling, and assessment of preferential exosome cellular distribution

NSC-34 cells differentiated for 4 DIV were cocultured with N9 cells in RPMI medium for 24 h, at 37°C in a humidified atmosphere of 5% CO2. We used the normal proportion of microglia and neurons in the CNS (ratio 1:1) (Silva et al., 2011).

In the coculture model, the N9 microglial cells were plated in coverslips with paraffin wax feet, as described by Phatnani et al. (2013). The coverslips containing microglia were placed inverted over the layer of wt or mSOD1 NSC-34 MNs and maintained separated from such layer by the paraffin spots, thus avoiding direct contact between the two types of cells. Cells were plated in the same proportion (1:1) and maintained in coculture for 24 h. At the end of incubation, exosomes in the supernatant of NSC-34 (wt or mSOD1) with N9 cocultures were isolated as described above. To obtain PKH67 labeled fluorescent exosomes, the isolated exosomes were resuspended in PBS and mixed with an equal volume of PKH67 probe solution for 5 min at room temperature, using the PKH67 Fluorescent Cell Linker Kit (#MINI67, Sigma-Aldrich), as described by Dutta et al. (2014). Then, isolated exosomes were resuspended in RPMI medium and added either to N9 cells + wt NSC-34 MNs, or to N9 cells + mSOD1 NSC-34-MNs, using the above described cocultured system, for an additional period of 24 h and in a ratio of 1:1 (v/v). After incubation, NSC-34 MNs and N9 microglia were collected separately, and fixed with 4% (w/v) paraformaldehyde in PBS and cell nuclei were stained with Hoechst 33258 dye. UV and fluorescence images (original magnification: 630X) were acquired per sample by using Zen 2012 (blue edition, Zeiss) software.

### Interaction of exosomes from wt and mSOD1 NSC-34 MNs with N9 microglia

N9 cells were plated for 24 h before incubation with exosomes from wt and mSOD1 NSC-34 MNs, at a concentration of 1 × 10^5^ cells/ml. Exosomes from NSC-34 cells were resuspended in RPMI medium and incubated in N9 microglial cells, using a fixed ratio of 1:1. To assess exosome internalization by N9 cells, exosomes were labeled with PKH67, as previously described.

To evaluate the effects produced by exosomes on N9 microglia, we incubated the cells in RPMI medium in the absence (control), or in the presence of exosomes, either from wt NSC-34 MNs, or from mSOD1 NSC-34 MNs. N9 microglia responses were evaluated at 2, 4, and 24 h. These different time-points of incubation were accomplished to evaluate the effects produced by an early (2 and 4 h) and a lasting (24 h) period of exosome interaction with naïve N9 microglia. At the end of each incubation period, medium free of cellular debris was collected to assess the released soluble factors. Attached cells were: (i) fixed for 20 min with freshly prepared 4% (w/v) paraformaldehyde in PBS for immunocytochemical studies or to detect PKH67 labeled fluorescent exosomes; (ii) fixed with Fixing Solution for cellular senescence assays; (iii) used to extract total RNA using TRIzol® reagent, according to the manufacturer's instructions; or (iv) collected in Cell Lysis Buffer (Cell Signaling Beverly, MA, USA) plus 1 mM phenylmethylsulfonyl fluoride (PMSF, Sigma) for western blot analysis.

### N9 microglia phagocytosis assay

To evaluate the phagocytic ability of N9 microglia, cells were incubated with 0.0025% (v/v) fluorescent latex beads (#L1030, Sigma-Aldrich), diameter 1 μm, for 75 min at 37°C. For immunofluorescence detection, N9 cells were fixed for 20 min with freshly prepared 4% (w/v) paraformaldehyde in PBS and a immunocytochemical technique was performed as usually in our lab for microglial cells (Caldeira et al., 2014; Cunha et al., 2016). N9 microglia were immunostained with rabbit anti-ionized calcium-binding adaptor molecule 1 (Iba1) (1:250, #019-19741, Wako), and nuclei were counterstained with Hoechst 33258 dye (blue). UV and fluorescence images of ten random microscopic fields (original magnification: 400X) were acquired per sample using an AxioCam HR camera adapted to an AxioScope A1® microscope (Zeiss, Germany), and Zen 2012 (blue edition, Zeiss) software. The number of cells with ingested beads and total cells were counted using ImageJ software to determine the percentage of phagocytosing cells (Silva et al., 2010).

### Determination of senescent-like positive N9 microglia

Activity of senescence-associated beta-galactosidase (SA-β-gal) was determined as a biomarker of microglia-like senescence by using the Cellular Senescence Assay Kit (#KAA002RF, Merck Millipore, Darmstadt, Germany), according to the manufacturer's instructions. Nuclei were counterstained with hematoxylin (Merck). The number of total cells was counted in 10 microscopic fields with ImageJ software (original magnification: 400X) acquired to observe the complete well using Leica IM50 software and Leica DFC490 camera (Leica Microsystems, Wetzlar, Germany), adapted to an AxioSkope HBO50 microscope (Zeiss). The number of turquoise stained microglia (SA-β-gal-positive cells) was counted to determine the amount of senescent cells relatively to the total cell number (percentage) (Caldeira et al., 2014).

### Detection of NF-κB activation

For immunofluorescence detection of nuclear factor-kappa B (NF-κB) translocation, cells were fixed as above and a standard immunocytochemical technique (Fernandes et al., 2006) was carried out using a rabbit anti-p65 NF-κB subunit antibody (1:200, #sc-372, Santa Cruz Biotechnology®, CA, USA). N9 microglial nuclei were stained with Hoechst 33258 dye. UV and fluorescence images of ten random microscopic fields (original magnification: 400X) were acquired as indicated for phagocytosis assay. NF-κB positive cells were identified by the ratio between the mean gray value of the nucleus and the mean gray value of the whole cell, using ImageJ software. A threshold was defined for each individual experiment and cells above that value were considered positive for NF-κB. Positive cells and total cells were counted to determine the percentage of NF-κB positive nuclei.

### Quantification of nitrite levels

Levels of nitric oxide (NO) were estimated by measuring the concentration of nitrites (${\text{NO}}_{2}^{-}$), a product of NO metabolism, in the extracellular media of N9 cells incubated in the absence, or in the presence of exosomes released by wt and mSOD1 NSC-34 cells. Extracellular media, free from cellular debris, were mixed with Griess reagent [1% (w/v) sulfanilamide in 5% H_3_PO_4_ and 0.1% (w/v)N-1 naphtylethylenediamine, all from Sigma-Aldrich, in a proportion of 1:1 (v/v)] in 96-well tissue culture plates for 10 min in the dark, at room temperature, as we published (Vaz et al., 2015). The absorbance at 540 nm was determined using a microplate reader. A calibration curve was used for each assay. All samples were measured in duplicate.

### Assessment of gelatinases (MMP-2 and MMP-9) by gelatin zymography

Activities of MMP-9 and MMP-2 were determined in the N9 extracellular media after incubation in the absence and in the presence of exosomes released by wt and mSOD1 NSC-34 cells by performing a SDS-PAGE zymography in 0.1% gelatin-10% acrylamide gels, under non-reducing conditions, as usual in our lab (Silva et al., 2010). Briefly, after electrophoresis, the gels were washed for 1 h in a solution containing 2.5% Triton-X-100 to remove SDS and to renature the MMP species in the gel and then incubated at 37°C to induce gelatin lysis (buffer: 50 mM Tris pH 7.4, 5 mM CaCl_2_, 1 μM ZnCl_2_) overnight. Gels were then stained with 0.5% Coomassie Brilliant Blue R-250 (Sigma-Aldrich) and destained in 30% ethanol/10% acetic acid/H_2_O (v/v). Gelatinase activity, detected as a white band on a blue background, was measured using computerized image analysis (Image Lab™, Bio-Rad).

### Quantitative real time-PCR

Total RNA was extracted from exosome-treated N9 microglia using TRIzol® (LifeTechnologies, Carlsbad, CA, USA), according to manufacturer's instructions. Total RNA was quantified using Nanodrop ND-100 Spectrophotometer (NanoDrop Technologies, Wilmington, DE, USA) and conversion to cDNA was performed with SensiFAST™ cDNA synthesis (#BIO-65054, BIOLINE, London, UK). Quantitative RT-PCR (qRT-PCR) was performed on a 7300 Real Time PCR System (Applied Biosystems) using a SensiFAST™ SYBR® (Hi-ROX, #BIO-92002/S, BIOLINE). qRT-PCR was accomplished under optimized conditions: 50°C for 2 min and 95°C also for 2 min, followed by 40 cycles at 95°C for 5 s and 62°C for 30 s. In order to verify the specificity of the amplification, a melt-curve analysis was performed, immediately after the amplification protocol. Non-specific products of PCR were not found in any case. Results were normalized to β-actin and expressed as fold change. The sequences used for primers are represented in Table S1 (Supplementary Material). Relative miRNA concentrations were calculated using the ΔΔCT equation. RNA inside exosomes was extracted using miRCURY Isolation Kit - Cell (#300110, Exiqon, Vedbaek, Denmark). For miRNA analysis, conversion of cDNA was achieved with the universal cDNA Synthesis Kit (#203301, Exiqon), as described by Cardoso et al. (2012) and currently implemented in our lab (Caldeira et al., 2014; Cunha et al., 2016), following manufacturer's recommendations. The miRCURY LNA™ Universal RT miRNA PCR system (#203403, Exiqon) was used in combination with predesigned primers (Exiqon), represented in Table S1 (Supplementary Data), using SNORD110 as reference gene. The reaction conditions consisted of polymerase activation/denaturation and well-factor determination at 95°C for 10 min, followed by 50 amplification cycles at 95°C for 10 s and 60°C for 1 min (ramp-rate 1.6°/s). Relative miRNA concentrations were calculated using the ΔΔCT equation. All samples were measured in duplicate.

### Western blot

Cells were collected in Cell Lysis Buffer, as usual in our lab (Vaz et al., 2015). Briefly, total cell extracts were lysed for 5 min on ice with shaking, collected with cell scrapper, and sonicated for 20 s. The lysate was then centrifuged at 14,000 g for 10 min at 4°C, and the supernatants were collected and stored at −80°C. Protein concentration was determined using a protein assay kit (Bio-Rad, Hercules, CA, USA) according to manufacturer's specifications. Then, equal amounts of protein were subjected to SDS-PAGE and transferred to a nitrocellulose membrane. After blocking with 5% (w/v) nonfat milk solution, membranes were incubated with primary antibody mouse anti-HMGB1 (1:200, from Biolegend) diluted in 5% (w/v) BSA overnight at 4°C, followed by the secondary antibody goat anti-mouse HRP-linked (1:5,000, sc-2005, Santa Cruz Biotechnology®) diluted in blocking solution. Chemiluminescence detection was performed by using LumiGLO® reagent (Cell Signaling) and bands were visualized in the ChemiDoc^TM^ XRS System (Bio-Rad). The relative intensities of protein bands were analyzed using the Image Lab^TM^ analysis software (Bio-Rad).

### Statistical analysis

Results of at least seven independent experiments were expressed as mean ± SEM. Comparisons between the different parameters evaluated in wt and mSOD1 NSC-34 MNs were made via one-tailed Student's *t*-test for equal or unequal variance, as appropriate. In addition, we have performed unpaired *t*-test with Welch's correction when the variances were different between 2 groups. Comparison of more than two groups was done by one-way ANOVA followed by multiple comparisons Bonferroni *post-hoc* correction using GraphPad Prism 5 (GraphPad Software, San Diego, CA, USA). *P*-values of 0.05 were considered statistically significant.

## Results

### mSOD1 NSC-34 MNs and their derived exosomes show increased levels of miR-124

Lately, miRNAs are emerging as potent fine-tuners of neuroinflammation and reported to be dysregulated in ALS (Koval et al., 2013; Butovsky et al., 2015). However, the contribution of individual miRNAs to neurodegeneration and neuroinflammation in ALS disease remains to be elucidated. We decided to investigate alterations on specific inflamma-miRs in the mSOD1 NSC-34 MNs and in their derived exosomes, using for comparison wt cells and their correspondent exosomes.

**Figure 1:**
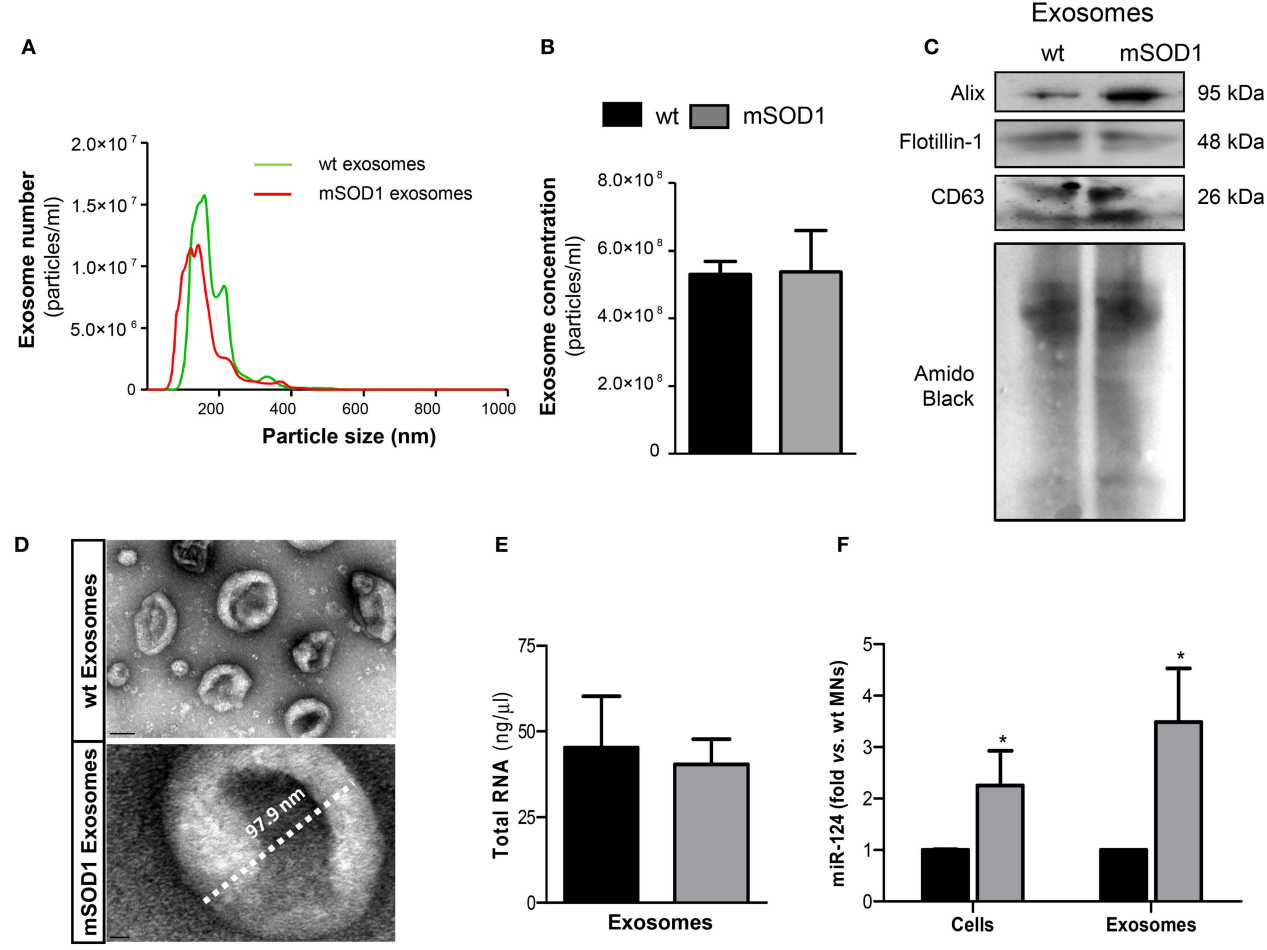
**Exosomes released by wild-type (wt) SOD1 NSC-34 motor neurons (MNs) and by those mutated in G93A (mSOD1) show similar number, size and total RNA content, but only mSOD1 NSC-34-derived exosomes display elevated expression of microRNA (miR)-124, thus recapitulating the donor cell**. Exosomes were isolated from the extracellular media of NSC-34 cells, either human wild-type SOD1 (wt MNs) or mutated in G93A (mSOD1 MNs), after 4 days *in vitro* differentiation, as described in methods. **(A,B)** Evaluation of the nanoparticles (exosomes) size and density by NTA indicates that the majority of vesicles from MNs have diameter ~130 nm, with no differences between wt and mSOD1 NSC-34 MNs in terms of particle concentration. **(C)** Western blot analysis indicates the presence of common exosome markers (Alix, Flotillin-1, and CD63). **(D)** Representative images obtained by transmission electron microscopy (TEM) of exosomes are depicted evidencing cup shape morphology and protein clusters. **(E,F)** RNA was extracted from cells and exosomes to evaluated microRNA (miRNA) expression. Quantification of total RNA **(E)** revealed no differences between samples from wt and mSOD1 NSC-34 MNs, while **(F)** miRNA profile show an increase only in miR-124 in both mSOD1 cells and in their derived exosomes. Results are mean (± SEM) from at least five independent experiments. Differences between mSOD1 NSC-34 MNs and wt NSC-34 MNs in cells and exosomes were obtained by the two-tailed Student's *t*-test with Welch's correction. ^*^p < 0.05 vs. respective wt MNs (Control). Scale bar represents 100 nm in exosomes from wt NSC-34 MNs and 10 nm in mSOD1 NSC-34 MNs.

Cellular differentiation was induced and exosomes were isolated from extracellular media after 4 DIV, to induce SOD1 accumulation, as previously reported (Vaz et al., 2015). Recent data from our group, obtained by dynamic light scattering and TEM, indicated that the nanoparticle size of exosomes in the extracellular media of NSC-34 cells was approximately of ~140 nm (Vaz et al., 2016). We have additionally performed a more detailed characterization by NTA analysis, having observed an average diameter size of ~130 nm and a concentration of ~5.3 × 10^8^ particles/ml (Figures 1A,B), independently of being originated from wt or mSOD1 NSC-34 MNs. Western blot analysis showed the presence of exosomal marker proteins, namely Alix, Flotillin-1, and the tetraspanin CD63 in lysates obtained after the exosomal isolation procedure (Figure 1C). In addition, exosomes isolated from wt and mSOD1 cells exhibited the characteristic cup-shape morphology by TEM (Figure 1D). It is important to refer that similar total RNA concentrations were found for exosomes isolated from wt or mSOD1 cells (Figure 1E). When compared to their wt counterpart, the mSOD1 MNs revealed an increased cargo in miR-124 (Figure 1F), which is the predominant miRNA in the brain, namely in neurons (Sun et al., 2015). No detectable amounts of miR-146a or miR-155 were found in either of them.

Since exosomes are known to contain miRNAs that can be transferred into recipient cells causing changes in their functionality (Valadi et al., 2007; Alexander et al., 2015), we decided to evaluate the miRNA expression in the MN-derived exosomes. Interestingly, exosomes from mSOD1 MNs evidenced to be enriched in miR-124, recapitulating their cells of origin, and again no expression of miR-146a or miR-155 was detected in either type of exosomes.

### Exosomes from wt and mSOD1 NSC-34 donor cells are similarly disseminated in the recipient N9 microglia, and when added to NSC-34+N9 cocultures they preferentially distribute in N9 microglial cells

**Figure 2:**
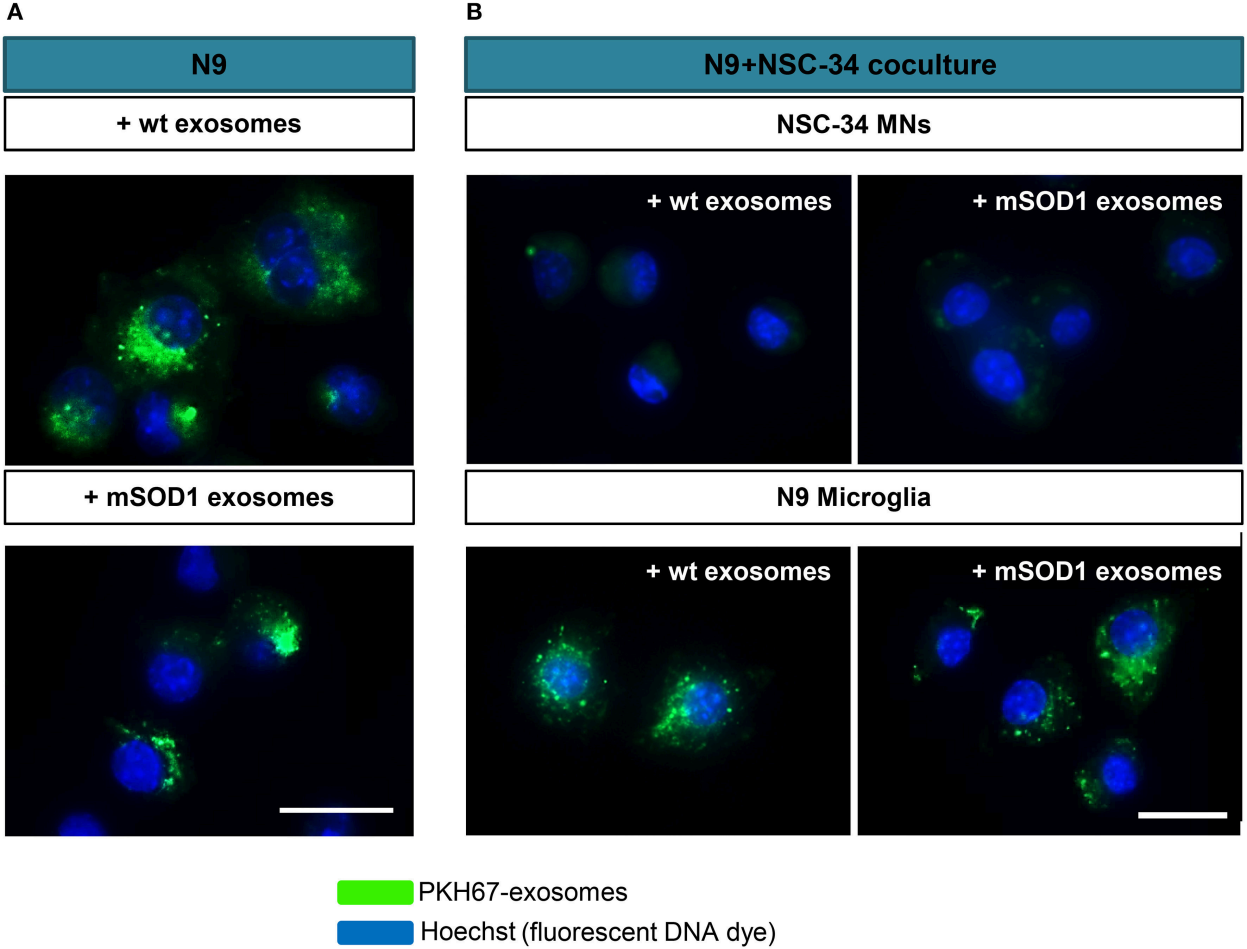
**Exosomes from NSC-34 motor neurons (MNs), either wild-type (wt) or mutated in G93A (mSOD1) are equally distributed in N9 microglia, which also revealed to be the preferential recipient cells for exosomes derived from MNs+N9 cocultures. (A)** Exosomes were isolated from NSC-34 MNs (wt and mSOD1), stained with the PKH67 Fluorescent Cell Linker Kit (in green) and incubated with N9 microglial cells for 24 h, and representative results of one experiment show the distribution of NSC-34-derived exosomes in monocultured N9 cells. **(B)** In parallel studies, exosomes were isolated from NSC-34 and N9 cells cocultures, stained as before and incubated for 24 h in a fresh NSC-34+N9 coculture and representative results of one experiment indicate the preferential distribution of exosomes in N9 microglia, as compared with NSC-34 MNs, when in the coculture system. Nuclei were stained with Hoechst dye (in blue). Scale bar represents 20 μm.

NSC-34 cells were demonstrated to uptake extracellular vesicles from a muscle cell line (C2C12) (Madison et al., 2014) and toxic exosomes were shown to be transferred from mSOD1 astrocytes to MNs (Basso et al., 2013). To determine whether exosomes released from mSOD1 and wt NSC-34 cells after 24 h incubation were similarly transferred into N9 microglial cells, we fluorescently labeled exosomes with PKH67, as previously described (Figure 2A). No differences were found in such distribution. To further understand whether mixed exosomes in the supernatant of NSC-34(wt)+N9 and of NSC-34(mSOD1)+N9 cocultures were preferentially captured by MNs or by N9 microglia, we isolated exosomes from the culture medium, labeled them with PKH67, incubated the exosomes with matched NSC-34(wt)+N9 and NSC-34(mSOD1)+N9 cocultures, and assessed the distribution of PKH67-labeled exosomes in either one of the cells. In Figure 2B, it is clearly shown that N9 microglia are the preferential recipient cells for the mixed exosomes released from both donor cell types. Indeed, intracytoplasmic green exosomes are only visible in N9 microglia, indicating that these cells are more likely to incorporate and to be functionally influenced by exosomes, as compared to NSC-34 MNs.

### Increased HMGB1 gene expression in mSOD1 NSC-34 MNs may contribute to its enhanced nuclear expression in the N9 microglia when cocultured with such cells

**Figure 3:**
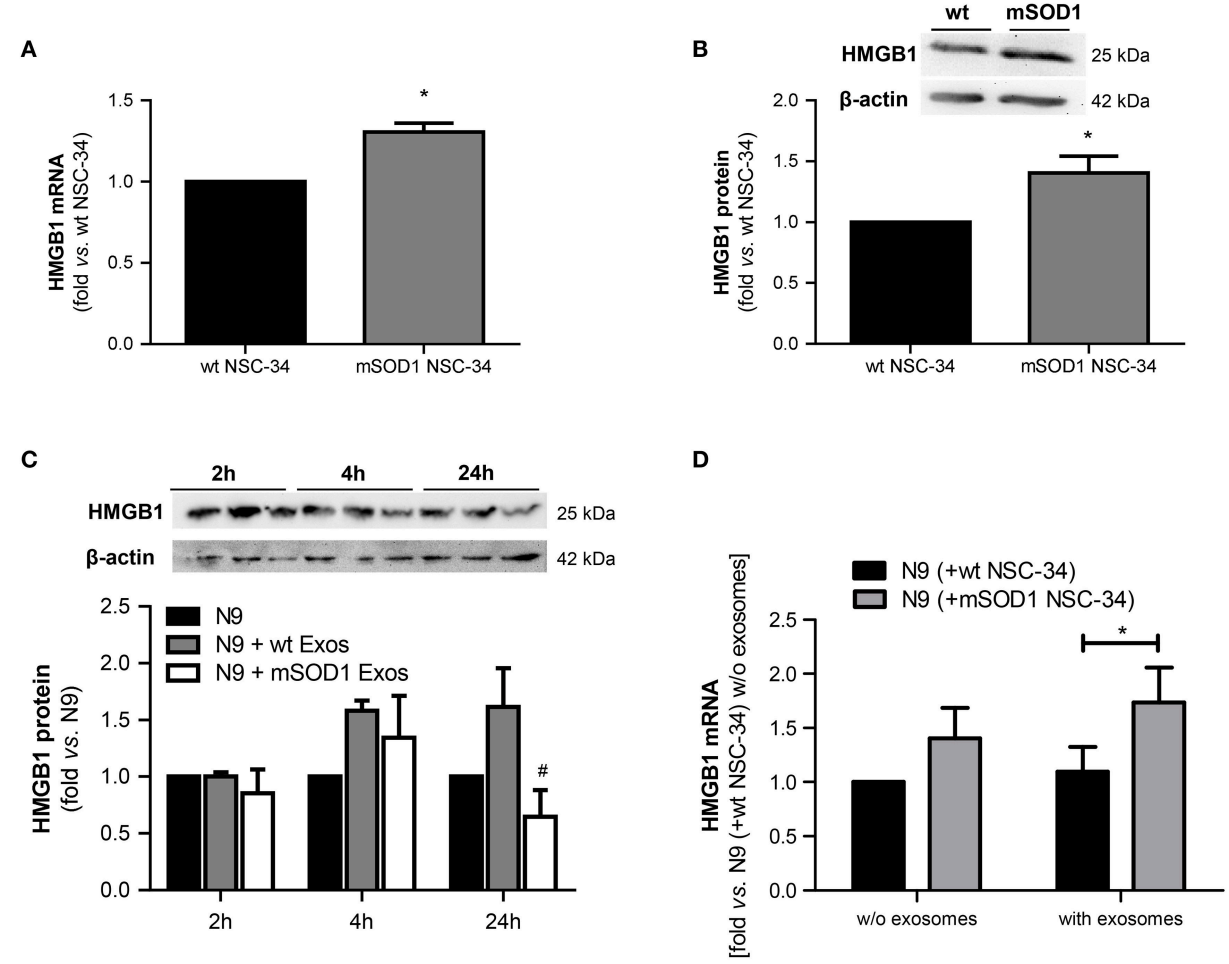
**HMGB1 upregulation is only observed in mSOD1 NSC-34 motor neurons (MNs) and in N9 microglia cocultured with MNs after surcharge with exosomes isolated from the coculture supernatants, mainly if containing mSOD1 MN-derived exosomes**. High mobility group box 1 (HMGB1) gene **(A)** and protein **(B)** expression was evaluated by qRT-PCR and Western Blot, respectively, in NSC-34 cells expressing either human wild-type SOD1 (wt MNs), or mutated in G93A (mSOD1 MNs), after 4 days *in vitro*. HMGB1 protein was also evaluated in **(C)** N9 cells-microglia incubated for 2, 4, and 24 h with exosomes (Exos) from wild-type (wt) NSC-34 MNs and mSOD1 NSC-34 MNs (N9+wt Exos and N9+mSOD1 Exos, respectively) or in **(D)** N9 cocultured with either wt or mSOD1 MNs, incubated or not with exosomes isolated from the media of a matched NSC-34-N9 coculture experiment, as indicated in methods. Results are mean (± SEM) from at least four independent experiments and are expressed as fold change vs. respective wt MNs. Differences between mSOD1 NSC-34 MNs and wt NSC-34 MNs were obtained by two-tailed Student's *t*-test with Welch's correction **(A,B)**. Differences between the three different groups at each time point were obtained by one-way ANOVA followed by Bonferroni *post-hoc* correction **(C,D)** ^*^p < 0.05 vs. respective wt NSC-34 MNs.^#^*p* < 0.05 vs. treatment with exosomes from wt NSC-34 MNs.

HMGB1 is a ubiquitous nuclear protein that is increasingly expressed and released by injured neurons and activated microglia (Gao et al., 2011; Brites and Vaz, 2014; Cunha et al., 2016). To evaluate whether mSOD1 MNs that were shown to be dysfunctional (Vaz et al., 2015) expressed increased HMGB1 and influenced the expression of HMGB1 in N9 microglia, we assessed its expression levels in both wt and mSOD1 NSC-34 MNs, as well as in N9 microglia when in coculture with NSC-34 MNs, in the absence and in the presence of a surcharge of exosomes isolated from the coculture supernatant (Figure 3).

We observed up-regulated HMGB1 gene and protein expression in mSOD1 NSC-34 MNs, as compared to wt cells (Figures 3A,B). To note, however, that the exosomes *per se* did not produce noticeable alterations in the N9 microglia HMGB1 gene expression in the absence of mSOD1 NSC-34 MNs (data not shown). The decrease observed in N9 microglia at 24 h incubation with mSOD1 exosomes (Figure 3C), suggests either degradation/cleavage of the protein or its release into the cell supernatant. In addition, although not significant, we observed a slight elevation in HMGB1 mRNA levels in the N9 microglia exposed to mSOD1 NSC-34 MNs. Significant increase in the HMGB1 gene expression was, however, obtained in N9 cells cocultured with mSOD1 MNs (without cell contact) surcharged with exosomes isolated from a 24 h matching coculture system (Figure 3D, *p* < 0.05), emphasizing secretome relevance in the signaling mechanisms underlying HMGB1-induced microglial activation.

Therefore, we next decided to evaluate if the internalization of wt and mSOD1 NSC-34 MN-derived exosomes in N9 microglia caused changes in the cell dynamic properties and function, determined by specific cell polarization phenotypes.

### Exosomes released by mSOD1 NSC-34 MNs lead to persistent NF-κB activation and early production of inflammatory mediators in the recipient N9 microglia

The proinflammatory transcription factor NF-κB has been shown to activate numerous molecules and factors, and to be critical in the regulation of neuroinflammation-associated disease pathogenesis, (Shih et al., 2015). Thus, we evaluated whether mSOD1 exosomes were able to activate NF-κB in N9 cells, a process implicated in MN death in ALS (Frakes et al., 2014).

**Figure 4:**
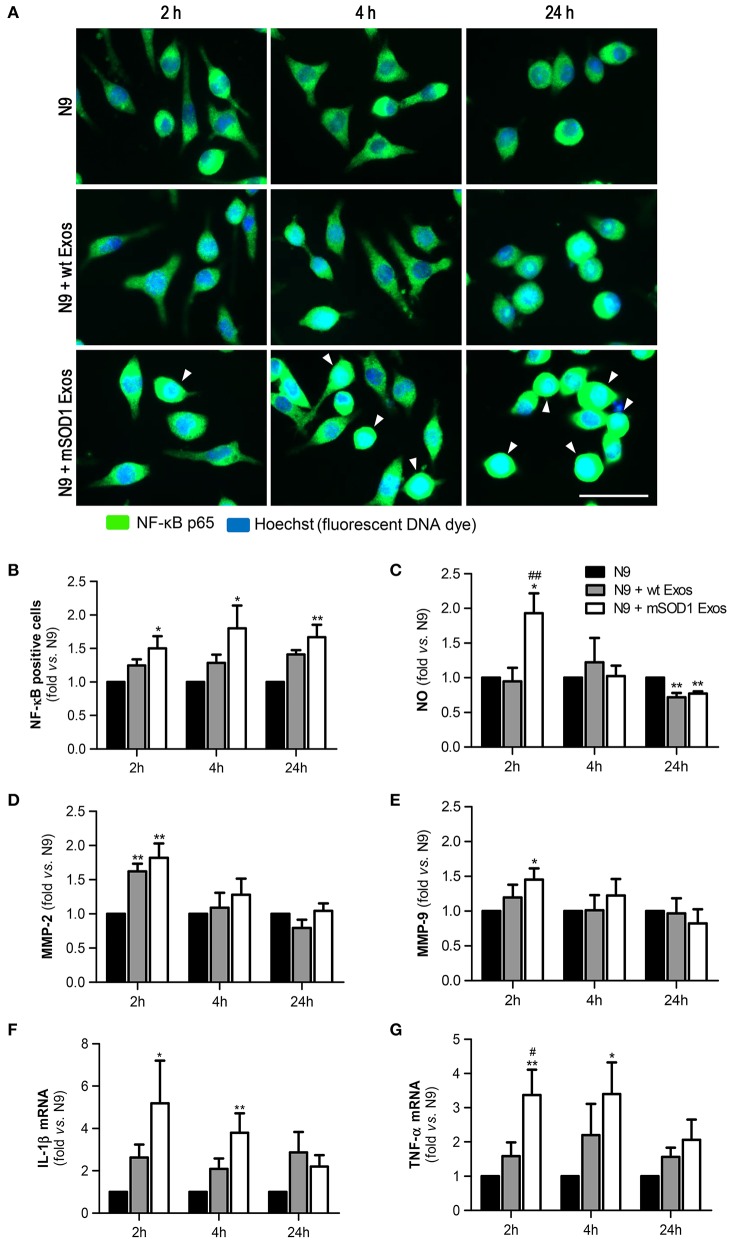
**Exosomes derived from NSC-34 motor neurons (MNs) mutated in G93A (mSOD1) lead to sustained NF-κB activation and acute production of inflammatory mediators in the recipient N9 microglia**. N9 cells were incubated for 2, 4, and 24 h with exosomes (Exos) from wild-type (wt) NSC-34 MNs and mSOD1 NSC-34 MNs (N9+wt Exos and N9+mSOD1 Exos, respectively), as indicated in methods.Non-treated cells were considered as control. **(A)** Representative results of nuclear factor kappa B (NF-κB) translocation into the nucleus and **(B)** number of NF-κB positive cells after interaction of exosomes with microglia. **(C)** Nitric oxide (NO) production was assessed by Griess reaction. **(D,E)** Activation of metalloproteinases (MMP)-9 and MMP-2, respectively, was assessed by gelatin zymography assay. **(F,G)** Relative tumor necrosis factor-α (TNF-α) and interleukin-1β (IL-1β) mRNA levels, respectively, was determined by qRT-PCR in total RNA. The fluorescence intensity of cells was quantified using the ImageJ software. Results are mean (± SEM) from at least seven independent experiments and are expressed as fold change relatively to non-treated N9 microglia. Differences between the three different groups at each time point were obtained by one-way ANOVA followed by Bonferroni *post-hoc* correction. ^*^p < 0.05 and ^**^p < 0.01 vs. non-treated cells; ^##^*p* < 0.01 vs. treatment with exosomes from wt NSC-34 MNs. Scale bar represents 40 μm.

We observed that although a slight effect was produced by exosomes derived from wt NSC-34 MNs on the NF-κB translocation into the nucleus, only those from mSOD1 NSC-34 MNs activated significantly and persistently (from 2 to 24 h incubation) the NF-κB signaling pathway (Figures 4A,B). This early and lasting NF-κB activation (Sen and Smale, 2010) suggest that distinct sets of genes are activated in N9 microglia upon interaction with exosomes released from ALS NSC-34 MNs.

We and others have previously shown that NO is a key player in MN degeneration in ALS (Drechsel et al., 2012; Vaz et al., 2015) and that increased generation of redox molecules (NO) and iNOS activation occurs in M1 polarized N9 microglia (Cunha et al., 2016). Increased NO production was observed after 2 h of incubation with exosomes only from mSOD1 MNs (Figure 4C). Such effect disappeared after 4 h incubation and even an inhibitory effect was produced by MN-derived exosomes at 24 h of incubation.

Activation of MMPs is another marker of neuroinflammation and elevation of MMP-9 and MMP-2 expression was observed in the spinal cord of SOD1G93A mice (Fang et al., 2010). Exosomes revealed to induce the MMP-2 activation whenever released from wt or mSOD1 MNs (Figure 4D). Intriguingly, only those from mSOD1 NSC-34 MNs were able to activate MMP-9 (Figure 4E), in accordance with our prior data showing such activation in mSOD1 NSC-34 MNs (Vaz et al., 2015). However, similarly to NO, this increase ceased over time, returning to basal levels.

Finally, we observed that the expression of the pro-inflammatory cytokines TNF-α and IL-1β was significantly upregulated and maintained until 4 h interaction, differently from the above mentioned inflammatory mediators, in cells exposed to exosomes from mSOD1 NSC-34 MNs, also disappearing after 24 h incubation (Figures 4F,G).

Based on these data we may assume that exosomes from the mSOD1 NSC-34 MNs transiently switch N9 microglia into a M1 polarized cell (Durafourt et al., 2012; Chhor et al., 2013). Since early or late NF-κB activation was shown to induce different sets of genes, by respectively encoding TNF-α, IL-8, MMP-9, or cell surface receptors, adhesion molecules and signal adapters (Tian et al., 2005), we next evaluated the effects produced on the expression of cell surface receptors.

### Exosomes from mSOD1 NSC-34 MNs lead to a delayed upregulation of receptors involved in N9 microglia response to stimuli

To determine whether late NF-κB activation in microglia treated with mSOD1 exosomes was associated with the increased expression of membrane surface receptors, like TREM2, RAGE, and TLR4, we evaluated their gene expression levels in a time-dependent manner. Indeed, microglia was shown to express multiple receptors able to efficiently respond to external stimuli (Pocock and Kettenmann, 2007).

**Figure 5:**
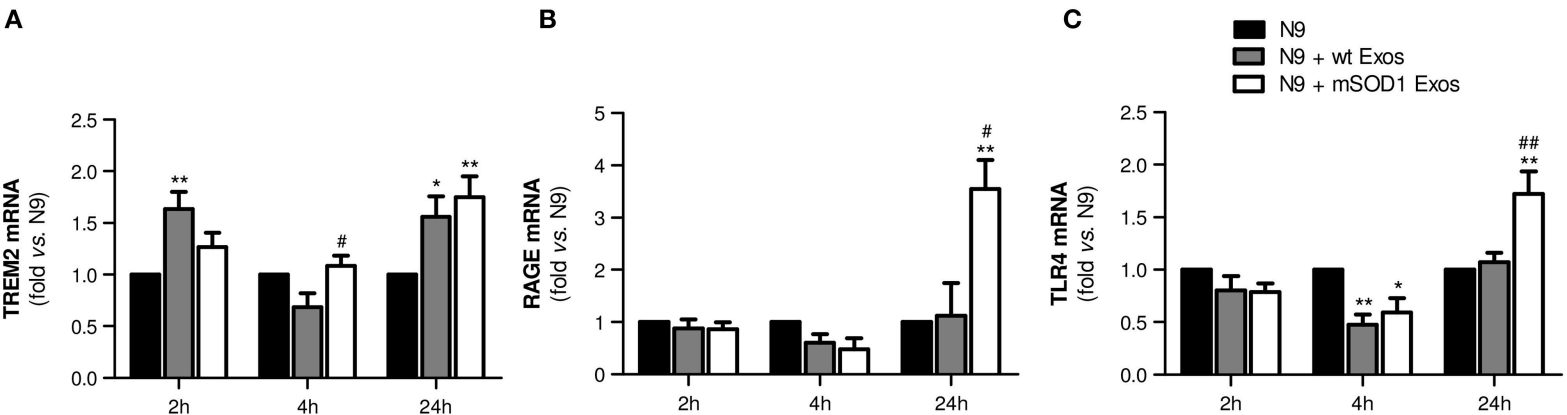
**Exosomes from NSC-34 motor neurons (MNs) mutated in G93A (mSOD1) lead to delayed upregulation of the receptors TREM2, RAGE and TLR4 in N9 microglia**. N9 cells were incubated for 2, 4, and 24 h with exosomes (Exos) from wild-type (wt) NSC-34 MNs and mSOD1 NSC-34 MNs (N9+wt Exos and N9+mSOD1 Exos, respectively), as indicated in methods. Non-treated cells were considered as control. Gene expression of **(A)** triggering receptor expressed on myeloid cells 2 (TREM2), **(B)** Receptor for Advanced Glycation Endproducts (RAGE) and **(C)** Toll-like receptor-4 (TLR4) was determined by qRT-PCR in total RNA. Results are mean (± SEM) from at least five independent experiments and are expressed as fold change relatively to non-treated N9 microglia. Differences between the three different groups at each time point were obtained by one-way ANOVA followed by Bonferroni *post-hoc* correction. ^*^p < 0.05 and ^**^p < 0.01 vs. non-treated cells; ^#^*p* < 0.05 and ^##^*p* < 0.01 vs. treatment with exosomes from wt NSC-34 MNs.

TREM2 receptor has been identified as a potential regulator of the microglial phenotype (Stefano et al., 2009) and found elevated in the spinal cord of ALS patients and SOD1G93A mice (Cady et al., 2014). As depicted in Figure 5A, increased expression of TREM2 gene in N9 microglia was evident after 24 h incubation with both wt NSC-34 MNs and mSOD1 MNs-derived exosomes, although some fluctuations were observed overtime. TREM2 overexpression has been related with suppression of neuroinflammation and microglia M2 polarization associated with increased phagocytic ability (Painter et al., 2015; Jiang et al., 2016).

RAGE is also a receptor found elevated in association with mSOD1 (Shibata et al., 2002). In the present study, it is clear its net elevation only in the N9 microglia treated for 24 h with exosomes from mSOD1 MNs (Figure 5B, *p* < 0.05 vs. wt NSC-34 MNs, and *p* < 0.01 vs. non-treated N9 microglia).

Besides RAGE, elevation of TLR4 was also identified in the spinal cord of sALS patients, mainly in glial cells (Casula et al., 2011). Both receptors play an important role in the regulation of innate and adaptive immunity during neuroinflammation. RAGE was recently indicated as enhancing TLR responses through binding and internalization of RNA (Bertheloot et al., 2016). Therefore, it was not surprising to find the same pattern of increased gene expression of TLR4 only in cells incubated for 24 h with exosomes released by mSOD1 NSC-34 MNs (Figure 5C).

At this point, our data indicate that exosomes from mSOD1 NSC-34 MNs determine an early inflammatory response on N9 microglia, which by releasing inflammatory mediators trigger the activation of RAGE/TLR signaling mechanisms and a second delayed stage of activation.

### Exosomes from mSOD1 NSC-34-MNs induce an early M1 polarization and heterogeneous (M1/M2) microglia subclasses at lasting times

In order to fully understand the effect of mSOD1 NSC-34-derived exosomes in N9-microglia phenotypic diversity, we searched for pro- and anti-inflammatory markers expressed in M1 and M2 microglial phenotypes (Freilich et al., 2013; Brites and Vaz, 2014; Cunha et al., 2016), respectively.

**Figure 6:**
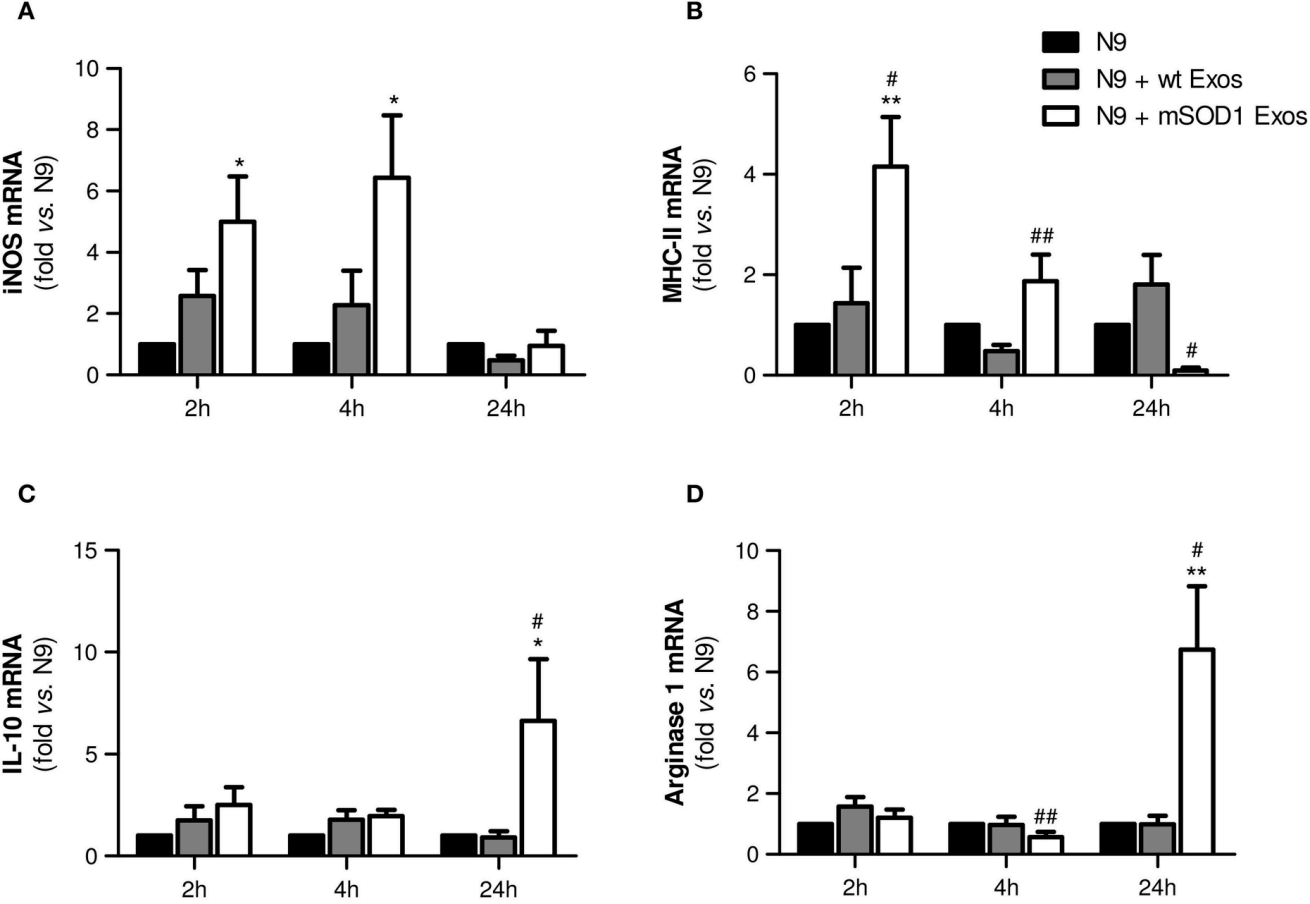
**Exosomes from NSC-34 motor neurons (MNs) mutated in G93A (mSOD1) trigger early upregulation of M1- and late expression of M2-markers in N9 microglia**. N9 microglia cells were incubated for 2, 4, and 24 h with exosomes (Exos) from wild-type (wt) NSC-34 MNs and mSOD1 NSC-34 MNs (N9+wt Exos and N9+mSOD1 Exos, respectively), as indicated in methods. Non-treated cells were considered as control. Relative mRNA levels of **(A)** inducible form of nitric oxide synthase (iNOS), **(B)** major histocompatibility complex-class-II (MHC-II), **(C)** interleukin-10 (IL-10), and **(D)** arginase-1 were determined by qRT-PCR in total RNA. Results are mean (± SEM) from at least eight independent experiments and are expressed as fold change relatively to non-treated N9 microglia. Differences between the three different groups at each time point were obtained by one-way ANOVA followed by Bonferroni *post-hoc* correction. ^*^p < 0.05 and ^**^p < 0.01 vs. non-treated cells; ^#^*p* < 0.05 and ^##^*p* < 0.01 vs. treatment with exosomes from wt NSC-34 MNs.

Data showed that exosomes from mSOD1 NSC-34 cells trigger upregulation of the M1-associated markers iNOS and MHC-II after 2 and 4 h incubation, but not after 24 h interaction (Figures 6A,B), suggesting that N9 microglial cells switch their polarization after continued interaction with the mSOD1 exosomes. Attenuated immune response with reduced MHC-II levels was observed at 24 h incubation, indicating that later, after activation, N9 microglial cells may downregulate MHC-II synthesis, as observed for dendritic cells (Villadangos et al., 2001). Indeed, the gene expression of M2-related markers, such as IL-10 and Arginase 1 (Figures 6C,D), was found substantially enhanced at this time after treatment with mSOD1 exosomes.

To study the role of exosomal miR-124, and other cargo contents, in producing microglia dynamic changes we evaluated the expression of two anti-inflammatory miRNAs (miR-146a and miR-124) and the proinflammatory miR-155, a recognized inducer of the M1 polarization found increased in ALS patients and models (Koval et al., 2013; Liu and Abraham, 2013; Butovsky et al., 2015) in N9 microglial cells after the transfer of mSOD1 exosomes.

We observed that a prompt reduction of calming miRNAs by NSC-34 MN-derived exosomes (Figures 7A,B–2 h incubation) was followed by a marked and moderate selective elevation of miR-124 and miR-155, respectively, by mSOD1 exosomes (Figures 7A,C–24 h incubation). Surprisingly, both wt and mSOD1 exosomes produced a delayed increase in miR-146a expression.

**Figure 7:**
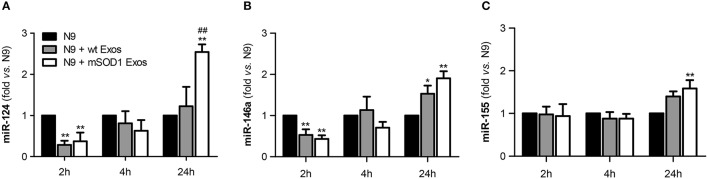
**Early decreased expression of calming microRNAs (miR-124 and miR-146a) is indicative of N9 microglia M1 phenotype, but their increase together with that of miR-155 suggests the coexistence of multiple activated phenotypes at 24 h**. N9 microglial cells were incubated for 2, 4, and 24 h with exosomes (Exos) from wild-type (wt) NSC-34 MNs and mSOD1 NSC-34 MNs (N9+wt Exos and N9+mSOD1 Exos, respectively), as indicated in methods. Non-treated cells were considered as control. **(A–C)** Relative miR-124, miR-146a, and miR-155 levels were determined by qRT-PCR in total RNA. Results are mean (± SEM) from eight independent experiments and are expressed as fold changes relatively to non-treated microglia. Differences between the three different groups at each time point were obtained by one-way ANOVA followed by Bonferroni *post-hoc* correction. ^*^p < 0.05 and ^**^p < 0.01 vs. non-treated cells; ^##^*p* < 0.01 vs. treatment with exosomes from wt NSC-34 MNs.

The immediate decrease in the N9 microglial miR-124 and miR-146 upon interaction with exosomes, indicative of M1 (pro-inflammatory) in opposite to M2 (alternative) microglia subtype, may justify the acute upregulation of inflammatory mediators previously observed (Figures 4, 6) for both wt (not significant) and mSOD1 NSC-34 MN-derived exosomes (at least *p* < 0.05). In contrast, the marked elevation of miR-124 at 24 h incubation in the N9 microglia treated with mSOD1 exosomes may derive, at least in part, from its increased content in MNs and in their derived exosomes that are collected by the cells, thus skewing M1 to M2a polarization (Veremeyko et al., 2013).

The upregulation of both calming and inflammatory miRNAs at 24 h, subsequent to the transfer of mSOD1 exosomes into the N9 cells, is indicative of induction of different polarized microglia subtypes, representing heterogeneous classes of activated N9 microglia, including both M1/M2 phenotypes. Influence of these diverse and simultaneous states on the variable rate of ALS progression surely deserves further investigation.

### Exosomes from mSOD1 NSC-34 MNs induce loss of N9 microglia phagocytic ability and cellular senescence

After identifying that exosomes released by mSOD1 NSC-34 MNs induce phenotypical alterations, we asked whether the N9 microglial cells were disturbed in one of their most relevant neuroprotective functions, the phagocytic ability. Also based on the elevation of miR-146a at 24 h incubation produced by both sorts of exosomes in the N9 microglial expression, we questioned if increased cell senescence was produced. Actually, miR-146a was already suggested as a marker of a senescence-associated proinflammatory status (Olivieri et al., 2013; Caldeira et al., 2014).

**Figure 8:**
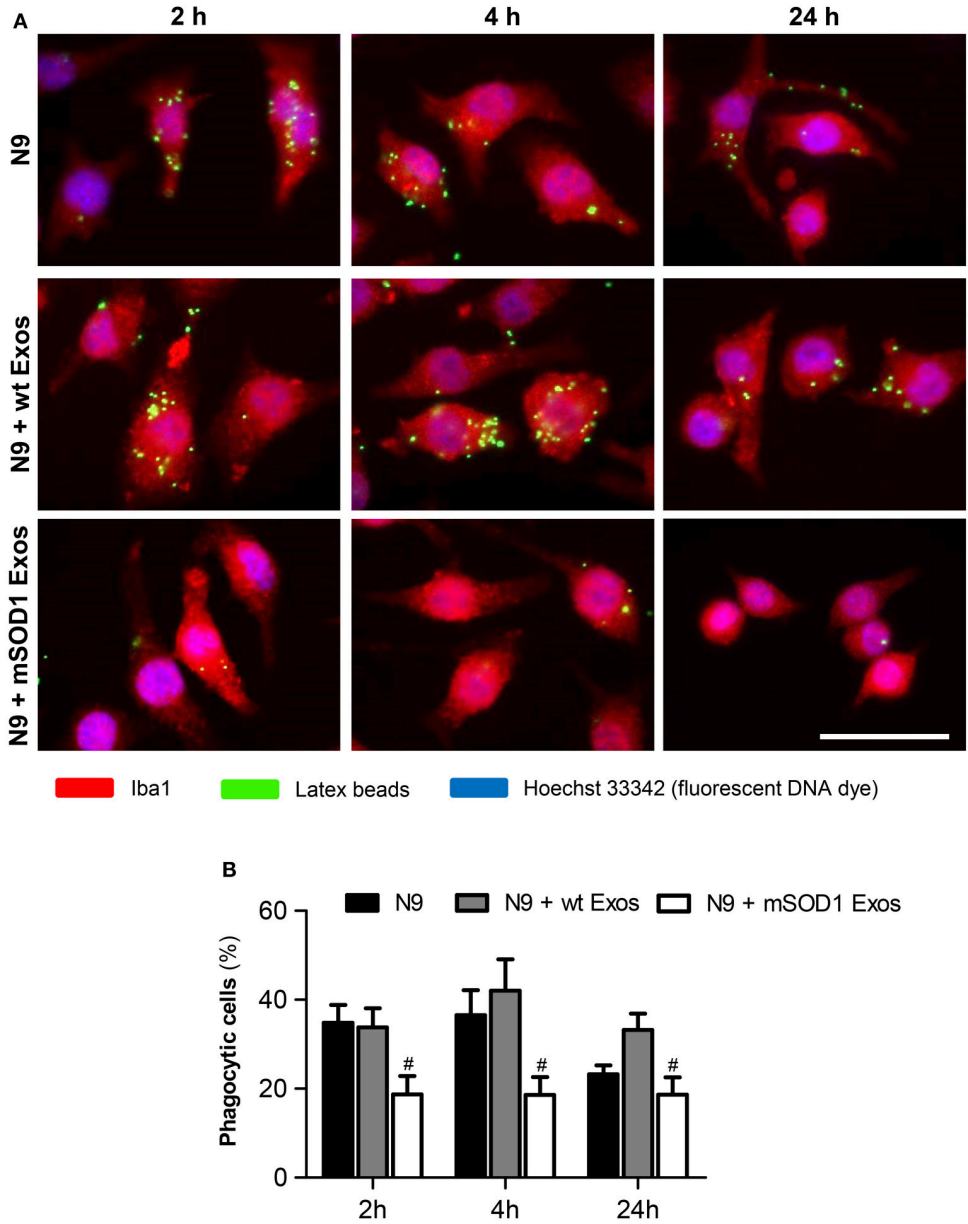
**Exosomes from NSC-34 motor neurons (MNs) mutated in G93A (mSOD1) determine a sustained and marked decrease in the N9 microglia phagocytic ability**. N9 microglial cells were incubated for 2, 4, and 24 h with exosomes (Exos) from wild-type (wt) NSC-34 MNs and mSOD1 NSC-34 MNs (N9+wt Exos and N9+mSOD1 Exos, respectively), as indicated in methods. Non-treated cells were considered as control. **(A)** Representative results of one experiment, showing engulfed latex beads (in green) by the Iba1 stained (in red) microglia with nuclei labeled by Hoechst dye (in blue). **(B)** Results are expressed as percentage of cells, relatively to the total number of microglia, showing ingested beads. Results are mean (± SEM) from eight independent experiments. Differences between the three different groups at each time point were obtained by one-way ANOVA followed by Bonferroni *post-hoc* correction. ^#^*p* < 0.05 vs. exosomes from wt MNs. Scale bar represents 40 μm.

As depicted in Figure 8, we observed a sudden and sustained decreased in the number of engulfed beads by N9 microglia upon interaction with mSOD1 exosomes, not noticed with those from wt NSC-34 MNs. Increased release of cytokines at 2 and 4 h incubation followed by upregulation of TLR4 after treatment with mSOD1 exosomes for 24 h, may account to this compromised N9 microglia function.

We have previously used the quantitative assay of SA-β-gal activity to evaluate the increase in the number of senescent-like microglia (Caldeira et al., 2014), as similarly proposed by other Authors (Debacq-Chainiaux et al., 2009). Results revealed that the percentage of positively stained cells increased after 24 h of incubation with exosomes from mSOD1 MNs, when compared with non-treated cells (Supplementary data, Figure S1). Therefore, and despite of causing less than 10% enhancement in the number of senescent-like N9 microglia, such occurrence may additionally contribute to microglia impaired function.

Overall these findings suggest that besides inducing microglia M1/M2 activation, exosomes from mSOD1 NSC-34 MNs also generate harmful effects by triggering the loss of microglia protective functions.

## Discussion

Understanding the role of exosomes, either in physiological or in pathological conditions, as well as their involvement in the spreading of disease, is essential for a complete comprehension of the intra and extracellular signaling events involved in ALS and as clues for the development of more effective therapeutic strategies in this pathology. Exosomes are nowadays considered as mediators of neuroinflammation (Gupta and Pulliam, 2014). We are the first in elucidating the effects of stably transfected mSOD1 NSC-34 MN-derived exosomes on the N9 microglia activation profile and on its functional properties. Previous studies demonstrated that toxic exosomes are transferred from mSOD1 astrocytes to MNs (Basso et al., 2013) and that exosome-dependent and independent mechanisms are involved in mSOD1 propagation (Grad et al., 2014a). It was lately hypothesized that extracellular vesicles are mainly released from live cells and from cells in the early dying process. It was proposed that within the recipient cell, the misfolded SOD1 species can template the misfolding of normally folded wt SOD1 (Silverman et al., 2016). In this work we established that exosomes derived from mSOD1 NSC-34 cells are enriched in miR-124 and responsible for N9 microglia activation and loss of function.

MiRNAs are genome encoded, short, non-protein coding RNA molecules that are expressed throughout the brain operating as fine tuners of post-transcriptional intricate events and involved in neuronal function and dysfunction (Saba and Schratt, 2010; Im and Kenny, 2012). Indeed, miRNAs are known to be crucial for neuronal differentiation and miR-124 was indicated to regulate hundreds of genes and to counteract astrocyte-specific route (Neo et al., 2014). When miR-124 is down-regulated, it triggers defective neuronal survival and reduced axonal outgrowth (Sanuki et al., 2011). Thus, overexpression of miR-124 was shown to be related with neuronal differentiation in neuroblastoma cell lines and embryonic stem cells (Krichevsky et al., 2006; Makeyev et al., 2007), and to contribute to neurite outgrowth (Yu et al., 2008), and neurogenesis (Visvanathan et al., 2007). MiR-124 was also found expressed in a subset of sensory neurons and suggested to have different functions and/or targets (Makeyev et al., 2007).

We evaluated the expression of specific inflamma-miRs in the mSOD1 NSC-34 cells. In contrast with the undetectable amounts of miR-146a and miR-155, we observed an upregulation of miR-124 in the mSOD1 NSC-34 MNs. Moreover, and similarly to the presence of miR-124a found in secreted exosomes from primary cortical neurons (Morel et al., 2013), we noticed that exosomes from mSOD1 NSC-34 MNs collected by ultra-centrifugation were enriched in miR-124, as well. Those Authors additionally documented that such exosomes were internalized by astrocytes where they modulated the astroglial glutamate transporter GLT-1. Here, we have observed that the exosomes released from NSC-34 MNs when incubated with N9 microglia and NSC-34 MNs, were preferentially collected by N9 microglia instead of being transferred into NSC-34 MNs. Previous studies have also evidenced the selective transfer of exosomes from oligodendrocytes to microglia (Fitzner et al., 2011). Interestingly, elevation of miR-124 in nerve terminals was associated to a decreased neurotransmitter release at the neuromuscular junction (Kye and Goncalves Ido, 2014), probably accounting to their dysfunction. Moreover, miR-124 upregulation was also demonstrated to be connected to a decreased capacity of cells to repair DNA strand breaks (Chen et al., 2015) and to be increased by stressful conditions (Sun et al., 2015). Clearly, the harmful or beneficial effects of miR-124 upregulation in ALS require further investigation, namely in terms of its transfer to microglia. Although with unknown biological significance in the periphery, its specific brain localization and presence in serum exosomes after acute ischemic stroke (Ji et al., 2016) is indicative of its promising potential as a biomarker of brain damage.

Spreading mechanisms are likely to underlie ALS disease progression based on the propensity of mutant SOD1 to misfold, on conditions that accelerate aggregation of wt SOD1 and on the interplay between affected neurons and their neighboring glial cells (Maniecka and Polymenidou, 2015). SOD1 cell-to-cell transmission may occur via both exosome-dependent and exosome-independent routes (Grad et al., 2014b). Indeed, these Authors demonstrated that NSC-34 cells stably transfected with mutant SOD1 release neurotoxic species of SOD1 that are transferred to naïve cells by macropinocytosis via conditioned medium transfer, either associated with exosomes (relatively efficient), or as protein-only aggregates. Interestingly, previous studies have shown that extracellular aggregated mSOD1 incubated for 24 h with microglia lead to increased ROS production and TNF-α release, and that the aggregates were internalized after 1 h incubation with minimal degradation after 24 h (Roberts et al., 2013). Since we observed that the exosomes released from NSC-34 MNs, when incubated with N9 microglia and NSC-34 MNs, were selectively transferred into N9 microglia, we decided to evaluate the temporal progression of the mechanisms leading to microglia activation by the mSOD1 MN-derived exosomes.

Previous studies in the spinal cord of SOD1G93A mice suggest that HMGB1 is not involved as a primary event in the MN death and that no changes occur relatively to its subcellular distribution in glial cells (Lo Coco et al., 2007). Further studies documented that increased expression of HMGB1, TLR4, and RAGE in reactive glial cells is observed in both gray (ventral horn) and white matter of the spinal cord from sALS patients (Casula et al., 2011). These Authors identified an elevated HMGB1 signal in the cytoplasm of glial cells and suggested that its release may be associated to the perpetuation of inflammation and necrosis of surrounding neurons due to inflammasome activation and secretion of proinflammatory cytokines, such as IL-1β and IL-18 (Lu et al., 2013; Baroja-Mazo et al., 2014). Recently, it was additionally showed that HMGB1 is a critical pathogenic molecule leading to neurite degeneration and innate-immune activation during Alzheimer's disease pathology (Fujita et al., 2016; Venegas and Heneka, 2017). Little is known about HMGB1 production and release by microglial cells, although we have shown that activated N9-microglia is able to secrete HMGB1 in response to the LPS-proinflammatory stimulus (Cunha et al., 2016) and to Aβ interaction (Falcão et al., 2017). HMGB1 also interacts with RAGE and TLR4, therefore extending the inflammatory cascade, while also promotes autophagy in detriment of apoptosis (Shen et al., 2013). Our results document an increased HMGB1 mRNA and protein levels in the mSOD1 NSC-34 MNs and in the N9 microglia cocultured with mSOD1 NSC-34 MNs in the presence of exosomes isolated from the extracellular media of such cultures, but not when N9 microglia is incubated with exosomes in the absence of NSC-34 MNs, suggesting that HMGB1 is released to the extracellular media after a prolonged incubation. Thus, we hypothesize that NSC-34 MN-derived soluble HMGB1 is necessary to induce N9 microglial HMGB1 enhanced expression, or that it is a consequence of a sustained microglial inflammatory status, after the release of proinflammatory cytokines and activation of RAGE and TLR4 receptors (Yu et al., 2006; Casula et al., 2011). Besides its delayed kinetic release, HMGB1-mediated production of proinflammatory cytokines requires the presence of these receptors, which we found to only be upregulated after 24 h of mSOD1 NSC-34 MN-derived exosomes interaction with naïve N9 microglia. The active secretion of HMGB1 into the extracellular milieu was documented to only begin 8–12 h after ligation to TLRs (Andersson and Tracey, 2011). In addition, previous studies indicated that the cytokine is a downstream and late mediator of inflammation that is released up to 1 week after admittance of patients with sepsis (Sunden-Cullberg et al., 2005).

TLR4 has been indicated to be involved in the pathological mechanisms of ALS disease, and blocking TLR4 with an antagonist extended the survival of the mSOD1 mice model (Lee et al., 2015). Recent evidences point out that the expression of RAGE is higher in the spinal cord of mSOD1 mouse model of ALS as compared with the wt one, and that pharmacological blockade of RAGE delays the progression of ALS and prolongs life span (Juranek et al., 2016). Here, we show for the first time that the expression of N9 microglial TLR4 and RAGE are enhanced in the N9 microglial cells upon the acceptance of exosomes from the mSOD1 NSC-34 MNs reinforcing the pathogenicity of such extracellular vesicles in ALS. In fact, protein levels of RAGE and its ligand HMGB1 were indicated to be elevated in ALS patients (Juranek et al., 2015).

TREM2 is another microglial receptor leading to downstream signaling cascades activation. It is implicated in multiple microglial transcriptional programs causing microglial phenotypes that do not fit the conventional M1/M2 paradigm and cell expansion (Poliani et al., 2015). TREM2 is thought to increase phagocytic activity and to suppress cytokine production (Chiu et al., 2013), while TREM2 p.R47H variant revealed to be a potent risk factor for sALS (Cady et al., 2014). Our data evidenced that exosomes *per se* induce a moderate, although significant late TREM2 gene upregulation. Controversial effects of increased expression of TREM2 protein were correlated with either apoptosis and decreased synaptic communication in Alzheimer's disease (AD) patient samples (Lue et al., 2015), or with an increased microglia ability to phagocytose, to inhibit Aβ proinflammatory responses and to rescue spatial cognitive impairment in the AD mouse model (Jiang et al., 2014). Therefore, potential benefits and harmful consequences of TREM2 upregulation require a further understanding on the still obscure microglia activation stages and their specific consequences on astrocytes and neurons.

Microglia were shown to induce MN death via the classical NF-kB pathway in ALS. Indeed, while NF-kB activation in astrocytes did not confer neuroprotection, its abolishment in microglia facilitated MN survival (Frakes et al., 2014). Based on this assumption we may conclude that the activation of the NF-kB pathway by the mutated exosomes add on microglia neurotoxicity toward MNs in ALS. The sustained NF-kB activation was paralleled by a loss in the phagocytic ability exclusively in N9 microglia exposed to mSOD1 exosomes. Previous studies demonstrated that TLR signaling inhibits the phagocytosis of apoptotic cells through NF-kB activation in microglia/macrophages (Feng et al., 2011; Deng et al., 2013) and that exosomes trigger the NF-kB signaling pathway (Matsumoto et al., 2005; Bretz et al., 2013). In the present study, such effect was mostly seen with mSOD1 exosomes, and the duration intended as determinant of different cellular responses (Bonnay et al., 2014): early release of proinflammatory mediators and late upregulation of cell surface receptors.

Neuroinflammation is a major component of ALS pathology, with activation and proliferation of microglia observed at sites of MN injury (Boillée et al., 2006). Our results show a marked increase of NO levels and MMP-2 and MMP-9 activation, upon a short exposure to exosomes from mSOD1 NSC-34 MNs. The increase in NO levels only occurred in cells exposed to mSOD1 exosomes, indicating their role as promoters of microglial oxidative stress. Extracellular mSOD1 activation of microglia was shown to be mediated through the CD14/TLR pathway, leading to activation of NF-κB, followed by upregulation of iNOS and release of NO from these cells (Zhao et al., 2010). Our data indicate a similar microglia activation triggered by mSOD1 exosomes from NSC-34 MNs, what is consentaneous with the TLR-dependent signaling pathways already proposed for exosomes (Bretz et al., 2013).

Elevation of MMP-9 activity was found primarily in ALS-associated NSC-34 MNs (Lim et al., 1996; Vaz et al., 2015). MMP-9 and MMP-2 secretion was suggested to be shed as membrane vesicle-associated components and to have a role in synaptic plasticity (Taraboletti et al., 2002; Sbai et al., 2008). Once ALS MNs release increased amounts of MMP-9 (Vaz et al., 2015), it may be hypothesized that increased levels of both MMPs in exosomes account for their subsequent release from the activated microglia. Accordingly, the expression of proinflammatory cytokines like TNF-α and IL-1β was also early upregulated in N9 microglia exposed to mSOD1 exosomes, and probably associated with the acute translocation of NF-kB to the nucleus and induction of genes involved in the production of proinflammatory mediators (Ghosh et al., 1998). Because activation of NF-kB in microglia was shown to induce gliosis and MN death, we may assume that exosomes from ALS NSC-34 MNs may have a role in neuroinflammation and neurodegeneration associated to ALS onset and progression (Frakes et al., 2014).

M1/macrophages/microglia have been associated to MN degeneration and ALS disease progression (Hooten et al., 2015; Lee et al., 2016), although a 50% reduction on reactive and proliferating microglia was initially shown to not influence neuronal damage (Gowing et al., 2008). Using established markers that allow the differentiation between M1 and M2 activated cells (Brites and Vaz, 2014), we observed that the M1-markers iNOS and MHC-II were early upregulated after transfer of mSOD1 exosomes into N9 microglial cells compatible with M1 polarization. Interestingly, we observed a delayed up-regulation of the M2-associated markers (Arginase 1 and IL-10) in N9 cells exposed for 24 h to exosomes from mSOD1 NSC-34 MNs, while levels of iNOS remained unchanged and MHC-II were even downregulated. This profile, together with sustained NF-κB activation and RAGE/TLR4/TREM2 upregulation at longer time-points suggest a switch of microglia phenotype from a classic M1 activated phenotype to a mixture of microglia subtypes that include M2 polarized cells. The precise harmful and still obscure role of microglia in ALS remains to be fully clarified, but may reside in the increased levels of miR-155 in the cell. Actually, Butovsky et al. (2015) found that miR-155 was overexpressed in the mSOD1 mouse, as well as in fALS and sALS patients, and that its targeting restored the dysfunctional microglia and attenuated disease progression in the mouse model. Other miRNAs besides miR-155 were also found upregulated in ALS microglia, such as miR-146b, miR-22, and miR-125b, thus strengthening the impact that miRNAs may have in modulating inflammatory genes and pathogenic mechanisms (Parisi et al., 2013).

Lately, exosomes released from activated cells were shown to contain inflammatory miRNAs, such as miR-146a, miR-155, and miR-21 among others (Xu et al., 2014; Alexander et al., 2015). We recently evidenced that miR-155 and miR-146a are increased in exosomes from LPS-induced M1 polarization of N9 microglia (Cunha et al., 2016). Other Authors (Alexander et al., 2015) also observed that these same miRNAs are released from dendritic cells within exosomes, pass between immune cells, negatively influencing (miR-146a) or promoting (miR-155) endotoxin-induced inflammation in mice. Therefore, we decided to evaluate the miRNAs associated with the modulation of the immune response (inflamma-miR), namely miR-155, miR-146a, and miR-124. Other miRNAs not indicated as directly implicated in microglia polarization were not considered in the present study. Our results identified their overall overexpression after 24 h incubation of the mSOD1 exosomes with N9 microglia. Therefore, we hypothesize that different microglia subpopulations may coexist with distinct roles that may include from neuroprotection to neurotoxic properties. The elevation of miR-155 is associated with RAGE overexpression and microglia M1 activation, while determine neurogenic deficits (Onyeagucha et al., 2013; Woodbury et al., 2015). In respect to miR-124 it was shown to promote microglia quiescence by diminishing M1 polarization and enhancing M2 phenotype (Ponomarev et al., 2011; Liu and Abraham, 2013), in particular the M2c microglia subset (Veremeyko et al., 2013). However, it is also a trigger of microglia functional maturity, at least during CNS development, where microglia evidence a reduced cellular motility and phagocytic ability (Svahn et al., 2016). Finally, miR-146a overexpression is found in M1, M2a, M2c, and senescent microglia subsets (Jiang et al., 2012; Cobos Jimenez et al., 2014). Our results further enhance the knowledge of the dysregulated miRNAs in ALS reinforcing miR-155 (Roberts et al., 2013), but also miR-146a and miR-124 among the most highly expressed in the microglia after internalization of mSOD1 NSC-34 MN-derived exosomes.

Here, we show that besides early and late activation processes and sustained activation of the NF-κB pathway, mSOD1 exosomes also trigger a substantial loss of the N9 microglia phagocytic ability, subsequently accompanied by an increased proportion of senescent-like microglia. Beneficial or detrimental consequences of microglial phagocytosis in tissue repair is a matter of controversy (Fu et al., 2014), but it has been claimed to be essential in the clearance of cellular debris, as well as in pathogenic organisms (Nakamura et al., 1999; Kloss et al., 2001). While the release of proinflammatory mediators is accepted as having a role in damage resolution, and chronic microglia activation as being associated with ageing and neurodegenerative diseases, much less attention has been paid to microglial phagocytosis, and to when such ability is reduced. Decreased phagocytic ability was demonstrated for senescent microglia in aging and in Alzheimer's disease models (Hickman et al., 2008; Zhu et al., 2011; Caldeira et al., 2014). While M1 microglia are often associated with acute inflammatory stimulus, M2 cells play a key role in tissue regeneration, promote phagocytosis and are designated as being protective. However, the distinction into M1/M2 subtypes is lately considered to be a simplification as represents the extreme states (Goldmann and Prinz, 2013). Actually, M2 (non-M1) activation state is considered to involve heterogeneous and functionally distinct macrophages/microglia (Roszer, 2015). Recent studies state that delayed cell clearance critically affects the dynamics of phagocytosis and suggest evaluation of phagocytic efficiency in neurological disorders (Abiega et al., 2016).

Taken together, the results obtained in this work indicate that exosomes released from mSOD1 NSC-34 MNs are enriched in miR-124 and are preferentially internalized by N9 microglia, causing a specific pattern of cell activation determined by early and late NF-κB and lasting decrease of the phagocytic ability. Acute response determines the increased production of proinflammatory mediators and cytokines. In such conditions microglia was shown to induce the formation of A1 reactive astrocytes with neurotoxic properties (Liddelow et al., 2017). Delayed activation is associated with enhanced expression of cell surface receptors and of miR-155, miR-146a, and miR-124. Therefore, exosomes from mSOD1 NSC-34 MNs initially polarize N9 microglia into the M1 proinflammatory phenotype, which may further enhance neuroinflammation and MN degeneration, together with a reduced ability to repair and maintain cellular homeostasis. However, with time, mSOD1 exosomes trigger different stages of activation leading to a miscellaneous population constituted by microglia expressing markers of M1, M2, and senescent states. In conclusion, exosomes released by the mSOD1 NSC-34 MNs have the potential to determine specific microglia subsets compatible with the previously assigned neurodegeneration-specific gene-expression profile found in the spinal cord microglia (Chiu et al., 2013). Despite the upregulation of the phagocytosis-related TREM2, TLR4 and RAGE surface receptors, microglia show a decreased ability to phagocytose and propensity to cell senescence upon exosome interaction, thus favoring the involvement of mSOD1 exosomes in the ALS onset and progression, whose precise mechanism of action requires additional studies.

## Author contributions

DB conceived the project. AV and DB planned and designed the experiments. SP isolated neuronal exosomes and performed all the evaluations on N9 microglia. MB performed the assays with cocultures. CC and AV participated in exosome isolation optimization procedure, vesicle characterization, and cargo evaluation. AV and DB interpreted experiments and wrote the paper with input from all authors. DB supervised all aspect and edited the manuscript.

### Conflict of interest statement

The authors declare that the research was conducted in the absence of any commercial or financial relationships that could be construed as a potential conflict of interest.

## Supplementary material

Figure S1 **Increased number of senescence-associated beta-galactosidase (SA-β-gal) expressing cells follows the exposure of N9 microglial cells to exosomes from mSOD1 NSC-34 MNs**. N9 microglial cells were incubated for 2, 4, and 24 h with exosomes (Exos) from wild-type (wt) NSC-34 MNs and mSOD1 NSC-34 MNs (MG+wt Exos and MG+mSOD1 Exos, respectively), as indicated in methods. Non-treated cells were considered as control. **(A)** Representative results of one experiment show senescent-like cells (SA-β-gal staining). **(B)** Results are expressed as the percentage of SA-β-gal positive cells (turquoise staining) relatively to the total number of microglia. Results are mean (± SEM) from at least five independent experiments. Differences between the three different groups at each time point were obtained by one-way ANOVA followed by Bonferroni *post-hoc* correction. ^*^*p* < 0.05 vs. non-treated cells. Scale bar represents 40 μm.

Table S1 **List of primer sequences used in qRT-PCR. (A)** Primers used in gene expression. **(B)** Primers used in microRNA expression.
