# Supplementary figures and images for: Exosomes from NSC-34 Cells Transfected with hSOD1-G93A Are Enriched in miR-124 and Drive Alterations in Microglia Phenotype
Source: Front Neurosci. 2017 May 17;11:273. doi: 10.3389/fnins.2017.00273 (PMC5434170; doi:10.3389/fnins.2017.00273)

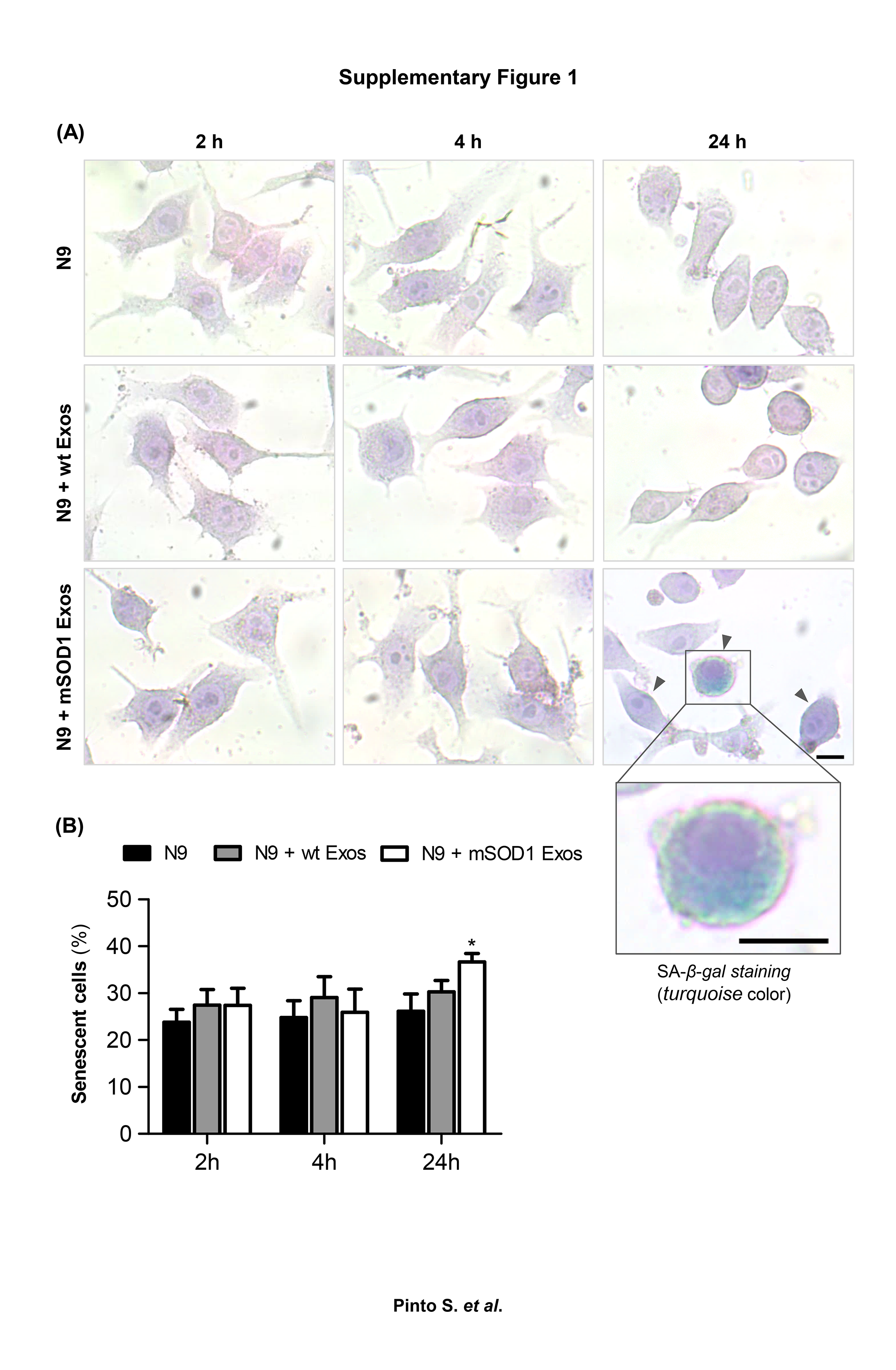

Supplement: Figure S1 — Increased number of senescence-associated beta-galactosidase (SA-β-gal) expressing cells follows the exposure of N9 microglial cells to exosomes from mSOD1 NSC-34 MNs. N9 microglial cells were incubated for 2, 4, and 24 h with exosomes (Exos) from wild-type (wt) NSC-34 MNs and mSOD1 NSC-34 MNs (MG+wt Exos and MG+mSOD1 Exos, respectively), as indicated in methods. Non-treated cells were considered as control. (A) Representative results of one experiment show senescent-like cells (SA-β-gal staining). (B) Results are expressed as the percentage of SA-β-gal positive cells (turquoise staining) relatively to the total number of microglia. Results are mean (± SEM) from at least five independent experiments. Differences between the three different groups at each time point were obtained by one-way ANOVA followed by Bonferroni post-hoc correction. *p < 0.05 vs. non-treated cells. Scale bar represents 40 μm. [file Image1.TIF]
