# Supplementary material for: Exosomes from NSC-34 Cells Transfected with hSOD1-G93A Are Enriched in miR-124 and Drive Alterations in Microglia Phenotype
Source: Front Neurosci. 2017 May 17;11:273. doi: 10.3389/fnins.2017.00273 (PMC5434170; doi:10.3389/fnins.2017.00273)
Supplement: Table S1 — List of primer sequences used in qRT-PCR. (A) Primers used in gene expression. (B) Primers used in microRNA expression. [file Table1.DOCX]

Supplementary Table

**Table S1 – List of primer sequences used in qRT-PCR. (A)** Primers used in gene expression. **(B)** Primers used in microRNA expression.

| **A** | **Gene** | **Sequence (5’-3’)** |
| --- | --- | --- |
|  | *TNF-α* | 5’-TACTGAACTTCGGGGTGATTGGTCC-3’ (fwr) |
|  |  | 5’-CAGCCTTGTCCCTTGAAGAGAACC-3’ (rev) |
|  | *IL-1β* | 5’-CAGGCTCCGAGATGAACAAC-3’ (fwr) |
|  |  | 5’-GGTGGAGAGCTTTCAGCTCATA-3’ (rev) |
|  | *HMGB1* | 5’-CTCAGAGAGGTGGAAGACCATGT-3’ (fwr) |
|  |  | 5’-GGGATGTAGGTTTTCATTTCTCTTTC-3’ (rev) |
|  | *RAGE* | 5’-CTGGTGGGACTGTGACCTTG-3’ (fwr) |
|  |  | 5’-TCTGCCTGTCATTCCTAGCTC-3’ (rev) |
|  | *TREM2* | 5’-AGCTACCCGCTACTGCAAAG-3’(fwr) |
|  |  | 5’-TCACTGCCAGGGGGTCTAAG-3’ (rev) |
|  | *TLR4* | 5’-ACCTGGCTGGTTTACACGTC-3’ (fwr) |
|  |  | 5’-GTGCCAGAGACATTGCAGAA-3’ (rev) |
|  | *Arginase 1* | 5’-CTTGGCTTGCTTCGGAACTC-3’ (fwr) |
|  |  | 5’-GGAGAAGGCGTTTGCTTAGTTC-3’ (rev) |
|  | *IL-10* | 5’-ATG CTG CCT GCT CTT ACT GA-3’ (fwr) |
|  |  | 5’-GCA GCT CTA GGA GCA TGT GG-3’ (rev) |
|  | *iNOS* | 5’-ACCCACATCTGGCAGAATGAG-3’ (fwr) |
|  |  | 5’-AGCCATGACCTTTCGCATTAG-3’ (rev) |
|  | *MHC-II* | 5’-TGGGCACCATCTTCATCATTC-3’ (fwr) |
|  |  | 5’-GGTCACCCAGCACACCACTT-3’ (rev) |
|  | *β-actin* | 5’-GCTCCGGCATGTGCAA-3’ (fwr) |
|  |  | 5’-AGGATCTTCATGAGGTAGT-3’ (rev) |

| **B** | **microRNA** | **Sequence (5’-3’)** |
| --- | --- | --- |
|  | miR-124 | 5’-UAAGGCACGCGGUGAAUGCC-3’ |
|  | miR-146a | 5’-UGAGAACUGAAUUCCAUGGGUU-3’ |
|  | miR-155 | 5’-CTCAGAGAGGTGGAAGACCATGT-3’ |
|  | SNORD110 | Reference gene |
